# Autonomic responses of premature newborns to body position and environmental noise in the neonatal intensive care unit

**DOI:** 10.5935/0103-507X.20190054

**Published:** 2019

**Authors:** Evelim Leal de Freitas Dantas Gomes, Camilla Malta dos Santos, Anelise da Costa Souza Santos, Aline Gomes da Silva, Mariza Aparecida Malaquias França, Dyele Souza Romanini, Manoela Cristina Veiga de Mattos, Andrea Fernanda Leal, Dirceu Costa

**Affiliations:** 1 Universidade Nove de Julho - São Paulo (SP), Brasil.; 2 Hospital do Mandaqui - São Paulo (SP), Brasil.

**Keywords:** Heart rate/physiology, Prone position, Infant, newborn, Autonomic nervous system, Noise

## Abstract

**Objective:**

Evaluate the physiological and autonomic nervous system responses of premature newborns to body position and noise in the neonatal intensive care unit.

**Methods:**

A quasi-experimental study. The autonomic nervous system of newborns was evaluated based on heart rate variability when the newborns were exposed to environmental noise and placed in different positions: supine without support, supine with manual restraint and prone.

**Results:**

Fifty premature newborns were evaluated (gestational age: 32.6 ± 2.3 weeks; weight: 1816 ± 493g; and Brazelton sleep/awake level: 3 to 4). A positive correlation was found between environmental noise and sympathetic activity (R = 0.27, p = 0.04). The mean environmental noise was 53 ± 14dB. The heart rate was higher in the supine position than in the manual restraint and prone positions (148.7 ± 21.6, 141.9 ± 16 and 144 ± 13, respectively) (p = 0.001). Sympathetic activity, represented by a low frequency index, was higher in the supine position (p < 0.05) than in the other positions, and parasympathetic activity (high frequency, root mean square of the sum of differences between normal adjacent mean R-R interval and percentage of adjacent iRR that differed by more than 50ms) was higher in the prone position (p < 0.05) than in the other positions. The complexity of the autonomic adjustments (approximate entropy and sample entropy) was lower in the supine position than in the other positions.

**Conclusion:**

The prone position and manual restraint position increased both parasympathetic activity and the complexity of autonomic adjustments in comparison to the supine position, even in the presence of higher environmental noise than the recommended level, which tends to increase sympathetic activity.

## INTRODUCTION

The autonomic nervous system (ANS) is part of the peripheral nervous system and is responsible for the automatic homeostasis of an organism. The ANS is subdivided into two systems that work in synergy: the sympathetic and parasympathetic nervous systems.^(1)^ In the first months of life, the ANS develops rapidly. The sympathetic nervous system starts developing at the beginning of gestation, and the parasympathetic nervous system develops more in the perinatal period. Thus, the development and control of the parasympathetic nervous system are susceptible to stimuli in the surrounding environment, such as excessive noise and body position.^(2,3)^ The first six months of the postnatal period are fundamental for the maturation of the parasympathetic nervous system.^(4)^

Noise, hypoxia and premature birth are common in neonatal intensive care units (ICUs) and are associated with increases in catecholamines and sympathetic activity in newborns.^(5)^ Premature newborns have greater difficulty decelerating their heartbeat in the occurrence of external noise than full-term infants.^(6)^ The external stimuli to which premature newborns are often subjected include pain, light and noise, which are stressors that cause instability with regard to physiological control. Body positioning is a common practice and is necessary for the development of premature newborns who are not able to resist the force of gravity, maintain body alignment on the midline or maintain a physiologic flexion position on their own, all of which are fundamental to adequate neurological development. Inadequate positioning is another source of stress for this population, affecting behavior and probably autonomic control. Candia et al.^(7)^ evaluated the salivary cortisol levels of premature newborns and found lower levels when the newborns were in the prone position than in other positions, thus associating this position with the reduction of stress.

Body positioning, autonomic control and environmental stress in the neonatal ICU occur simultaneously. According to Peng et al.,^(8)^ premature newborns exhibit more stress in the supine position than in the prone position, which is associated with better respiratory mechanics and lower stress. However, studies show that this position is associated with a greater incidence of sudden infant death, less heart rate variability (HRV) and greater sympathetic activity during sleep. The hypothesis is that with regard to clinical variables and ANS responses, awake premature newborns, such as those in the study by Peng et al.,^(8)^ who are subjected to habitual environmental stimuli in the neonatal ICU may benefit from the prone position and the supine position with manual restraint for flexion, thus maintaining the body on the midline in comparison to the supine position without support.

The aim of the present study was to evaluate the physiological and ANS responses of premature newborns to body position and noise in the neonatal ICU.

## METHODS

A quasi-experimental prospective study was conducted with a single group of newborns in which each child served as his/her own control for each position.

This study was conducted in accordance with the precepts stipulated in the Declaration of Helsinki and the Nuremberg Code as well as the norms governing research involving human subjects (Resolution 466/2012 of the Brazilian National Board of Health). The study received approval from the Human Research Ethics Committee of the *Hospital do Mandaqui* and *Universidade Nove de Julho* (certificate of approval: 1.613.732/2016).

The study was conducted at the neonatal ICU of the *Hospital do Mandaqui*. The sample was composed of 50 newborns with 32 to 40 weeks of corrected age who were born between gestational ages of 30 and 36 weeks. The sample calculation was performed from a pilot study with 15 newborns in which an analysis of the correlation between sympathetic activity and noise was conducted (r = 0.40). Using a two-tailed significance of p = 0.05, beta error of 0.20, and power of 80%, the study sample should have at least 47 newborns. Fifty were included to guarantee the calculated sample size.

### Inclusion criteria

Were included newborns with 32 to 40 weeks of corrected age; weight less than 2500g and more than 1100g (only low-weight and very-low-birth-weight infants were included to avoid excessive handling of extremely low-birth-weight infants and to guarantee sample homogeneity); clinical stability with weight gain in the previous 72 hours; more than 72 hours of life; and stage 3 or 4 on the Brazelton sleep/awake scale.^(9)^

### Exclusion criteria

Were not included in the sample neonates using invasive or noninvasive mechanical ventilation; in use of oxygen therapy; with clinical instability; with active infection; and refusal to sign informed consent by a legal guardian.

### Experimental procedure

#### Measurement of environmental noise in the neonatal intensive care unit

**Figure 1:**
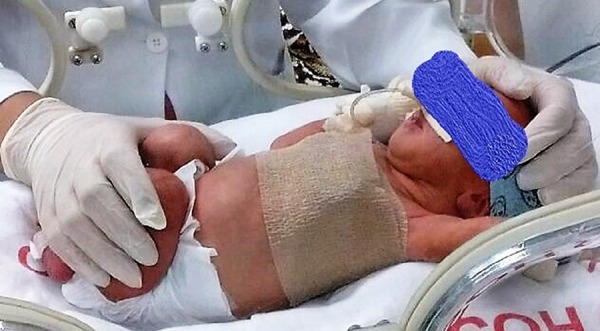
Manual restraint.

Noise was measured near each child's crib/incubator with the aid of a Sound Datalogger(tm) for 30 minutes during the normal care routine in the neonatal ICU.^(10)^ The data were subsequently analyzed using a specific software program and expressed in decibels (dB). When the infant was placed in the supine position with no support, in the dorsal decubitus position with restraint (Figure 1) or in the prone position, noise was measured minute by minute for 10 minutes in each position along with vital signs and HRV.

#### Heart rate variability

Sympathetic and parasympathetic activity was measured based on the analysis of HRV,^(10)^ which was determined using a Polar(tm) V800 portable monitor. The HRV indices analyzed in the time domain were the mean R-R interval (iRR), the standard deviation of all iRR (SDNN), the root mean square of the sum of differences between normal adjacent iRR (RMSSD), the percentage of adjacent iRR that differed by more than 50ms (pNN50) and the triangular index (total number of iRRs divided by the maximum height of the histogram of all iRRs). The HRV indices analyzed in the frequency domain were low frequency (LF: 0.04 to 0.15Hz), high frequency (HF: 0.15 to 0.4Hz) and the LF/HF ratio.^(11)^ Moreover, the following nonlinear indices were evaluated: standard deviation 1 (SD1), standard deviation 2 (SD2), approximate entropy (ApEn) and sample entropy (SampEn).^(12,13)^ The readings lasted 30 minutes: 10 minutes in the supine position without support, 10 minutes in the prone position and 10 minutes during manual restraint.

### Statistical analysis

The statistical analysis was performed using Minitab 14. The normality of the data was determined through a visual inspection of the curve and using the Shapiro-Wilk test. One-way analysis of variance (ANOVA) and Tukey's post hoc test were used for comparisons of the clinical variables and HRV for the different body positions. The level of significance was set to 5% (p < 0.05).

## RESULTS

The sample was composed of premature newborns with very-low birth weight, the majority of whom had APGAR scores of 9 in the fifth minute following birth. The newborns were hemodynamically stable with spontaneous breathing. The majority (56%) were born via a cesarean section procedure, and 44% were born via natural childbirth. Table 1 displays the characteristics of the sample.

**Table 1:** Characteristics of the study sample

Variables	N (%)	Minimum	Maximum	Mean	Standard deviation
Sex					
Male	26 (52)				
Female	24 (48)				
Gradational age (weeks)		30	36	32.6	2.3
30	11 (22)				
31	2 (4)				
32	7 (14)				
33	7 (14)				
34	11 (22)				
35	10 (20)				
36	2 (4)				
Chronological age (days)		3	28	9.23	9.5
APGAR (1 minute)		5	9	6.65	2.26
APGAR (5 minutes)		5	10	8.42	1.3
Birth body weight (g)		1,100	2,500	1,816.3	493.7
Study body weight (g)		1,260	3,300	2,123.4	671.5
Type of birth					
Cesarean	28 (56)				
Natural	22 (44)				

With regard to HRV variables, greater parasympathetic activity was found in the prone position than in the supine position in all domains, with a significant difference between the two positions in the frequency domain. In the analysis of nonlinear variables, manual restraint and the prone position promoted greater complexity in the autonomic adjustments than the supine position, which is considered positive from a physiological standpoint. Table 2 displays the HRV variables.

**Table 2:** Heart rate variability in the three positions

Variables	Supine	Restraint	Prone
Time domain			
Mean RR	466.1 ± 167.4	426.8 ± 52.7	433.9 ± 113.8
SDNN	27.02 ± 20.56	24.07 ± 13.52	29.52 ± 28.43
Mean HR	147.2 ± 25.1	142.20 ± 17.9	140.45 ± 20.7
RMSSD	9.87 ± 9.4	9.81 ± 7.3	11.19 ± 7.7
pNN50	0.62 ± 0.20	1.12 ± 4.4	1.45 ± 4.2
Frequency domain			
LF	82.24 ± 6.7*	80.49 ± 7.7	77.79 ± 9.4
HF	17.73 ± 6.7*	19.44 ± 7.6	21.57 ± 9.6
LF/HF	5.68 ± 32	5.08 ± 2.7	4.49 ± 2.9
Nonlinear			
SD1	6.08 ± 3.8	6.98 ± 5	7.54 ± 5.1
SD2	38.21 ± 28.9	33.41 ± 17.6	45.79 ± 52.9
ApEn	0.77 ± 0.28†*	0.96 ± 0.22	0.86 ± 0.29
SampEn	0.63 ± 0.36†*	0.88 ± 0.37	0.74 ± 0.36

RR - R-R interval; SDNN - mean standard deviation of all iRRs; HR - heart rate; RMSSD - root mean square of the sum of squared differences between iRRs; pNN50 - percentage of difference between adjacent normal R-R intervals greater than 50ms computed over the entire height of the histogram created by charting all R-R intervals; LF - low frequency; HF - high frequency; SD - standard deviation; ApEn - approximate entropy; SampEn: sample entropy;

* p < 0.05 prone *versus* supine,

† p < 0.05 supine versus restraint. Data are expressed as the mean and standard deviation.

**Figure 2:**
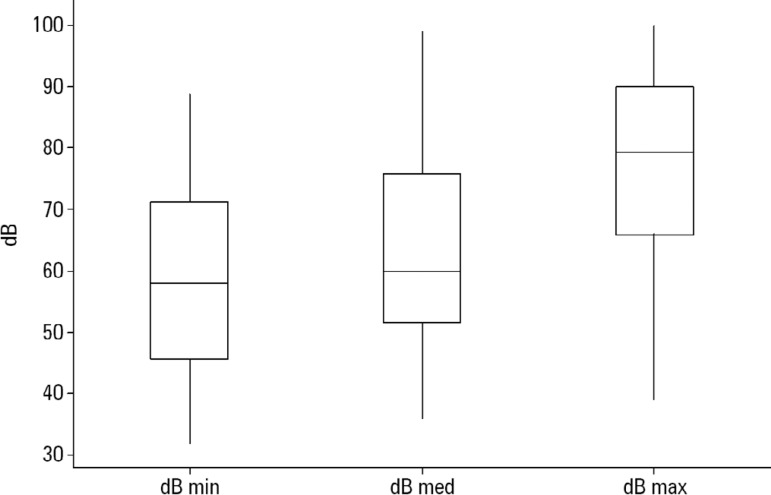
Decibels measured in the neonatal intensive care unit. min - minimum; med - medium; max - maximum.

Figure 2 illustrates the levels of noise in the neonatal ICU during the evaluation period, all of which surpassed the recommended level of 45dB.

**Figure 3:**
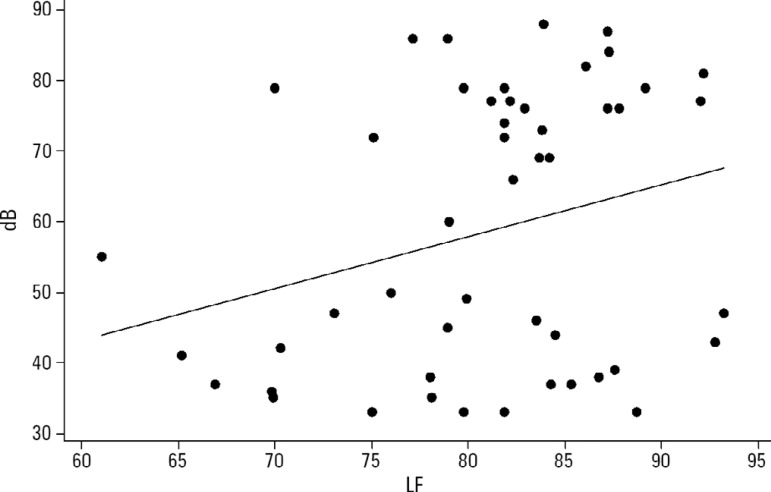
Correlation between noise and the sympathetic activity of the autonomic nervous system. HRV - heart rate variability; LF - low frequency.

A positive correlation was found between environmental noise and sympathetic activity in the newborns (Figure 3).

**Figure 4:**
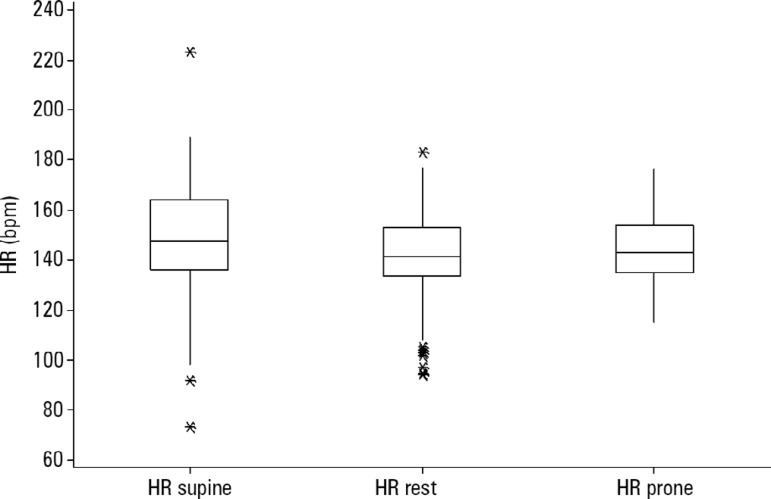
Mean heart rate (bpm) in different positions. HR - heart rate.

Figure 4 displays the mean heart rates (bpm) in the supine, prone and restrained positions. Higher means were found in the supine position than in the other two positions (*) (p < 0.05).

## DISCUSSION

The main finding of the present study was that the prone position promoted a reduction in stress behavior^(8,14,15)^ due to low visual stimulation as well as the known effects regarding improvements in respiratory mechanics.^(16)^ The reduction in visual stressors due to positioning promotes better self-regulation and homeostasis, with a consequent improvement in autonomic adjustments.^(8)^ Indeed, an increase in parasympathetic activity and good adjustment complexity demonstrated by the nonlinear HRV variables were found in the present study.

Stress provoked by the surrounding environment leads to an increase in energy expenditure in premature newborns, which exerts a negative impact on neurological integration as well as growth and development. The factors that contribute to environmental stress in the neonatal ICU are light, noise and handling. Previous studies^(10,17)^ found that the noise associated with the care offered in the neonatal ICU surpassed the limit of 45dB recommended by the American Academy of Pediatrics^(9)^ and that the noise could even surpass the maximum limit of safety (70dB). Team training for noise reduction in neonatal ICUs seems to be effective.

Similar results were found in the present study, in which the maximum noise level reached 90dB.^(18,19)^

Sahni^(3)^ evaluated the effects of the prone position on RR intervals with an electrocardiogram during sleep in newborns and found a reduction in parasympathetic activity, which is in disagreement with the present findings. However, differences were found in the cardiovascular dynamics between the states of sleep and wakefulness. In the present study, evaluations were made on newborns in stages 3 and 4 of the Brazelton scale.

The supine position is associated with greater arousal and greater energy expenditure than the prone position. Bell et al.^(20)^ measured energy expenditure by direct and indirect calorimetry and found that the energy expenditure was 10% lower in the prone position than in the supine position. In contrast, the prone position is associated with a greater risk of sudden infant death and should therefore not be used without supervision in the home environment. In a supervised environment, such as the neonatal ICU, the prone position can be adopted safely and offers numerous benefits, such as a position of flexion, better respiratory mechanics,^(21)^ better neuromotor organization and better autonomic adjustments.^(22,23)^

The prone position and lateral decubitus position are not safe because they may favor the rebreathing of expired gases and reduce the rate of body heat loss, thus leading to hypoxemia and hypercapnia and an increase in body temperature, predisposing the child to sudden infant death syndrome; therefore, these two position should not be used during sleep. Most studies evaluated a prone position during sleep; in the present study, newborns were awake (Brazelton 3 - 4), which is a divergent factor since there are alterations in the autonomic responses in different stages of sleep and wakefulness.^(24)^

Shepherd et al.^(25)^ evaluated cerebral blood flow and oxygen extraction in extreme and moderate newborns, and in the extreme population, there was a reduction in cerebral blood flow and a slight increase in oxygen extraction in the prone position, suggesting a greater need for the evaluation of position in this group than in the moderate group. In the group of moderate newborns and term infants,^(26)^ this change did not occur. We suggest strict evaluation of the clinic and the gestational age of the child when using the prone position. As previously stated, there are differences in adjustments during sleep and wakefulness, and when awake, the prone position seems to have benefits in terms of both autonomic adjustments and the reduction of stress.

The idea of offering postural comfort to premature newborns to alleviate stress is not new, but few studies have demonstrated the response to positioning by the ANS, and the population sizes analyzed have rarely surpassed 30 patients. Cong et al.^(27)^ evaluated the autonomic responses of newborns to pain, comparing infants in an incubator and those in kangaroo care (skin-to-skin contact), demonstrating that stability of the autonomic adjustments after pain was quickly achieved when the infant was in a comfortable position.

All the benefits of positioning in this population and the harmful effects of stress from the environment on the premature brain are widely known. This was one of the first studies to show that the apparently simple practice of positioning a newborn also improves autonomic adjustments despite the occurrence of normal noises in the neonatal ICU environment.

The development of the parasympathetic nervous system occurs in the perinatal period. Adequate stimuli result in optimal development of this system, whereas adverse stimuli can exert a negative effect on its development, with the possibility of future abnormalities in childhood and adulthood.^(4)^ The complexity of autonomic adjustments is a marker of good cardiovascular health, and this complexity is normally reduced in pathological situations, which demonstrates its importance.^(23,28-30)^

The limitations of this study are related to the evaluation of HRV because it is influenced by many factors; therefore, several inclusion criteria were adopted. In addition, the evaluation of HRV by itself with nonlinear methods, both in the time domain and in the frequency domain, is unable to characterize the dynamic parameters of HRV. The nonstationary character of HRV makes the use of stationary approaches to the study of HRV, as in the case of traditional methods, limited, although it provides us with information about the behavior of the ANS.

Another difficulty in studying this specific population is that invasive procedures should be avoided, thereby limiting more accurate evaluations. Finally, no medicines that might have interfered with the autonomic responses were adopted, so more rigorous inclusion criteria were adopted.

## CONCLUSION

The prone position and manual restraint for premature newborns increase both parasympathetic activity and the complexity of autonomic adjustments in comparison to the supine position, even in the presence of higher environmental noise than the recommended level, which tends to increase sympathetic activity.
